# Common Food Additives Inhibit Carbonic Anhydrase Activity

**DOI:** 10.1002/jbt.70444

**Published:** 2025-08-18

**Authors:** Ayça Aktaş Karaçelik, Murat Küçük, Semra Alkan Türkuçar, Şükrü Beydemir

**Affiliations:** ^1^ Department of Food Processing, Espiye Vocational School Giresun University Giresun Turkey; ^2^ Department of Chemistry, Faculty of Sciences Karadeniz Technical University Trabzon Turkey; ^3^ Department of Nutrition and Dietetics, Faculty of Health Sciences Alanya Alaaddin Keykubat University Alanya Antalya Turkey; ^4^ Department of Basic Pharmaceutical Sciences, Faculty of Pharmacy Anadolu University Eskişehir Turkey

**Keywords:** ADMET, carbonic anhydrase, enzyme inhibition, erythrosin B, food additives

## Abstract

Few studies about the effects of food additives on human enzymes exist. The effects of 20 food additives (12 colorants, 2 antioxidants, 3 sweeteners, 2 preservatives, and 1 acidity regulator) on bovine carbonic anhydrase (bCA) and especially human isoenzymes hCAI and hCAII isoenzyme, a highly vital enzyme, were studied for the first time. All the additives showed inhibition on human CA isoenzymes with IC_50_ values in 5‐5998 μM range. The higher inhibitions were detected in the colorants, Erythrosine B showing the highest inhibition (IC_50(bCA)_: 11 μM, IC_50(hCAI)_: 19 μM and IC_50(hCAII)_: 5 μM) at levels comparable with standard CA inhibitor sulfanilamide, while sweeteners showed low inhibition. BHT, a synthetic antioxidant, had higher inhibition compared to ascorbic acid. According to ADMET results, when the pharmacokinetic properties of the additives are considered, the only molecule with high gastrointestinal absorption is curcumin. The findings suggest that the health concerns caused by excessive consumption of foods containing additives may be related to CA inhibition. Food additive alternatives with no/lower CA inhibition should be sought for. Besides, Erythrosine B derivatives deserve investigation for new CA inhibitors.

## Graphical abstract

**Food additives inhibit carbonic anhydrases**

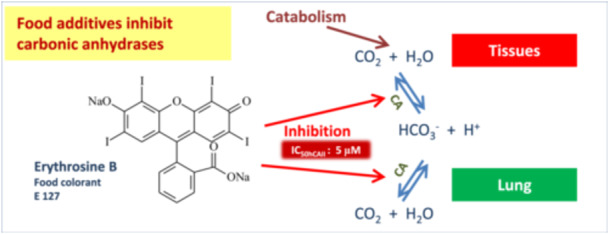

## 1 Introduction

Carbonic anhydrase (CA) (carbonate hydrolyase EC 4.2.1.1) is a metalloenzyme with a zinc (Zn^+2^) ion in its active site and characterized as a pH‐regulating enzyme in many cell types including erythrocytes. It is an important enzyme that catalyzes the reversible reaction of the hydration of carbon dioxide and the dehydration of bicarbonate in organisms [1].

The inhibition and activation effects of carbonic anhydrase enzyme activity by many chemicals and drugs have been investigated by scientists and reported in the literature [1, 2]. The enzyme is considered the target molecule for drugs in many tissues. Heteroaromatic sulfonamides, particularly acetazolamide, have been used for many years as the most potent organic inhibitors of the carbonic anhydrase enzyme. These inhibitors are currently being used as a guide in the treatment of glaucoma, epilepsy, and neurological disorders [3, 4], as anticancer [5], antitumor [6], antiobesity [7], antiepileptic [8, 9, 10], and in the development of positron emission tomography and magnetic resonance imaging, as well as diagnostic material in diagnosis, antiulcer, diuretic drugs [11]. Therefore, it has become very important in recent years to know the mechanism of inhibition of CA enzyme and discover new inhibitor compounds [12]. Natural or synthetic new CA inhibitors to be identified will be important in the discovery of new drug leads.

With the increasing trend in the production of processed foods in modern food technology, the use of food additives has accelerated. Food additives are mixtures of substances or substances which are not food alone but participate in food production, processing, storage, and packaging. They are classified according to their function. For example; colorants, substances that improve the color of the food or participate in the food for the purpose of coloring; antioxidants, substances that prolong the shelf life by preventing oxidation reactions; sweeteners, substances that give a sweet taste to the food, without sugar, preservatives, substances that increase the shelf life by preventing spoilage in food due to microorganisms and acid regulators, substances that alter or control the acidity or alkalinity of foods. The food additives that are commonly used in foods and selected in our study are given in Table 1.

**Table 1:** E codes, names, and usages of the food additives tested.

No	E code	Compound name	Usage
1	E131	Patent Blue V	Artificial colorant (triaryl methane class)
2	E129	Allura Red AC	Artificial colorant (red azo dye)
3	E102	Tartrazine	Artificial colorant (lemon yellow azo dye)
4	E124	Ponceau 4 R	Artificial colorant (mono azo dye)
5	E110	Sunset Yellow FCF	Artificial colorant (mono azo dye)
6	E133	Brilliant Blue FCF	Artificial colorant (triaryl methane class)
7	E151	Brilliant Black BN	Artificial colorant (diazo dye)
8	E127	Erythrosine B	Artificial colorant (xanthene class)
9	E123	Amaranth	Artificial colorant (azo dye)
10	E132	Indigo Carmine	Artificial colorant (indigoid class)
11	E100	Curcumin	Natural colorant
12	E120	Carminic acid	Natural colorant
13	E954	Saccharin	Artificial sweetener
14	E950	Acesulfame K	Artificial sweetener
15	E951	Aspartame	Artificial sweetener
16	E330	Citric Acid	Acidity regulator
17	E210	Benzoic acid	Preservative
18	E200	Sorbic acid	Preservative
19	E300	Ascorbic acid	Antioxidant
20	E321	Butylated hydroxytoluene (BHT)	Antioxidant

Studies regarding the effects of food additives on human enzymes are partially available, and there are few studies related to CA in the literature. There is a restricted number of studies concerning CA inhibition by food additives. Inhibition effects of several aromatic sulfonamides and azo dyes obtained by Sandmeyer reaction and di azo coupling on hCAII and hCAVII isoenzymes and also a number of diazenylbenzene sulfonamide compounds (azo dye derivatives containing phenol and amine moieties) on tumor‐associated CAIX and XII isoenzymes were investigated in vitro [13, 14]. In addition, studies on the effect of butylated hydroxytoluene (BHT) and butylatedhydroxyanisole (BHA) [15] used as synthetic antioxidants in foods, curcumin used as natural colorant [16, 17] saccharin, aspartame, and acesulfame K used as sweeteners [18, 19, 20, 21] and Allura Red AC used as artificial colorant on carbonic anhydrase enzyme activity are available in the literature [22, 23].

Moreover, several studies have been performed on the derivatives of the compounds such as saccharin and curcumin after the inhibition effect on the CA has been clarified [17, 19]. Similarly, in light of the information obtained from our studies, the food additive compound types acting on the hCA I and hCAII isoenzymes of CA will lead to the identification of new inhibitors and activators.

It is not known whether or not many of the food additives act on the CA enzyme activity. Studies to determine the effect of CA inhibition or activation on metabolism and the compounds that cause inhibition/activation are valuable. In this study, the effects of food additives on bovine carbonic anhydrase (bCA) and especially human isoenzymes hCAI and hCAII enzyme activities have been determined for the first time. The most novel aspect addressed in this paper is to compare the inhibitory properties of food additives on bCA and CA isoenzymes (hCAI and hCAII) and demonstrate safety profiles and drug similarity with in silico pharmacokinetic estimates (ADMET). This study will also give clues about whether the side effects associated with the consumption of foods containing food additives could be related to carbonic anhydrase enzyme inhibition. At the same time, the findings obtained from this study will enable the evaluation of the benefits and damages that may occur in the consumption of foods containing the components that highly inhibit the CA. Computer‐aided drug discovery (CADD) has emerged as a powerful tool to reduce the high costs associated with drug development. CADD leverages comprehensive genomic sequence data, protein structural information, and small molecule libraries to enhance drug discovery processes. It plays a key role in identifying target proteins, screening potential ligands, and predicting their absorption, distribution, metabolism, excretion, and toxicity (ADMET) properties [24].

This study aimed to investigate the effects of 20 different food additives (12 colorants, 2 antioxidants, 3 sweeteners, 2 preservatives, and 1 acidity regulator) on the bovine carbonic anhydrase enzyme (bCA) and human carbonic anhydrase isoenzymes (hCAI and hCAII) purified by affinity column chromatography. %CA inhibition and IC_50_ values of food additives were determined using CA esterase activity in all samples. The type of inhibition of Erythrosine B, which also showed inhibition effect on hCAI isoenzyme purified from human erythrocytes, was also determined. Additionally, ADME‐Toxicology analysis was performed for 20 different food additives using the SwissADME database [25]. The logP values, molecular weight, H‐bond acceptor and donor numbers, bioavailability score, molecular polar surface area, binding to plasma proteins, CYP2D6 inhibition, absorption in the human intestinal system and passage through the blood brain barrier of the molecules were investigated.

## 2 Materials and Methods

### 2.1 Chemicals

All food additives, Sepharose 4B, *p*‐nitrophenyl acetate (*p*‐NPA), protein assay reagents, and the reagents for electrophoresis were obtained from Sigma (Sigma‐Aldrich GmbH, Steinheim, Germany). All other chemicals were analytical grade and purchased from Merck.

### 2.2 Blood Supply and Hemolysate Preparation

The human blood used in the study was obtained from the blood bank of Karadeniz Technical University Medical Faculty Farabi Hospital, whose final use date was expired. An ethics committee certificate for human blood use (protocol number: 2015/119) was obtained. Bovine blood was taken fresh from a slaughterhouse and the separation process was started without delay.

Fresh blood was filled into 10 mL centrifuge tubes to separate the erythrocytes and centrifuged at 2500 rpm for 15 min. Plasma and leukocyte coats were removed. The erythrocytes were washed three times with isotonic NaCl solution and hemolysed with 1.5 volumes of ice‐cold water and then centrifuged at 20,000 rpm for 40 min (Beckman Coulter Allegra 121 64 R) at 4°C to remove erythrocyte cell membranes, and then the pH of hemolysate was adjusted to 8.7 with solid Tris and stored at +4°C until introduction to the affinity column for enzyme purification.

### 2.3 Preparation of Affinity Gel

Purification technique used in the preparation of affinity gel was applied in many previous studies [26, 27]. After activation of the free hydroxyl groups of Sepharose 4B with cyanogen bromide (CNBr), covalent attachment of tyrosine was performed. Finally, the route of binding of sulfanilamide with diazo‐coupling was followed. Tyrosine was used as the extension arm of the affinity gel, and sulfanilamide was used as a specific inhibitor of carbonic anhydrase to keep the enzyme in the column.

### 2.4 Purification of Human Erythrocyte Carbonic Anhydrase Isozymes and Bovine Carbonic Anhydrase Enzyme by Affinity Column Chromatography

The pH adjusted hemolysate (100 mL) was then subjected to affinity column [Chromatography system: column: 25 cm (Sigma), bed volume: 20 mL; peristaltic pump (Pharmacia LKB Pump‐1, U.S.A.), and fraction collector (Pharmacia LKB RediFrac, U.S.A.)] pre‐equilibrated with 25 mM Tris‐HCl/0.1 M Na_2_SO_4_ (pH: 8.7) for the purification of carbonic anhydrase enzymes. After the hemolysate loading was completed, wash buffer (25 mM Tris‐22 mM Na_2_SO_4_, pH: 8.7) was sent to the system to remove all undesirable compounds from the column. The human erythrocyte carbonic anhydrase isozymes hCAI and hCAII were eluted with 50 mM Na_2_HPO_4_ ‐ 1 M NaCl, pH: 6.3, and 0.1 M CH_3_COONa ‐ 0.5 M NaClO_4_, pH: 5.6, respectively (flow rate: 20 mL/h, fraction volume: 5 mL). The bovine enzyme was eluted with 0.1 M CH_3_COONa ‐ 0.5 M NaClO_4_ (pH: 5.6) buffer system. The protein elution by affinity column chromatography was monitored by absorbance measurement at 280 nm.

### 2.5 Hydratase Activity Determination

**Figure 1:**
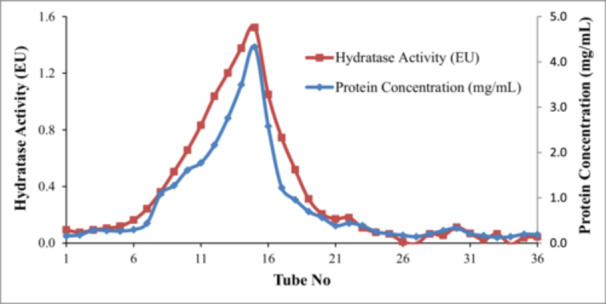
CA hydratase activity and protein content graphs of the purified bCA enzyme in the fractional collection tubes.

**Figure 2:**
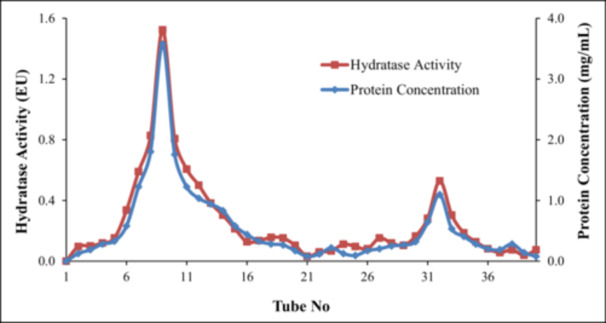
CA hydratase activity and protein content graph of purified hCAI and hCAII isoenzymes in the collection tubes.

Carbonic anhydrase activity was assayed by measuring hydratase activity according to the method of Wilbur‐Anderson, in which the time for the decrease of medium pH from 8.2 to 7.4 resulting from H^+^ production from the hydration of CO_2_ to carbonic acid was determined [26]. The reaction mixture contained a mixture of 100 μL of the enzyme and 2400 μL of 2.5 mM HEPES (pH: 8.8) buffer and 50 μL sample finally mixed with 2500 μL saturated aqueous CO_2_ solution. The control test was performed by preparing the mixture without enzyme. All the measurements were recorded as triplicate. The activity was expressed as percentage inhibition of CA activity by using the pH reduction times observed with blank (no enzyme) and positive control (with enzyme). The method was used to determine the carbonic anhydrase activity in the collection tubes in affinity chromatography (Figures 1 and 2).

### 2.6 Esterase Activity Determination

Carbonic anhydrase activity was also assayed by following esterase activity according to the method defined by Verpoorte et al. with slight modification [28, 29, 30]. The enzyme hydrolyzes the substrate, *p*‐nitrophenyl acetate, to *p*‐nitrophenol or *p*‐nitrophenolate. The change in absorbance was measured spectrophotometrically at room temperature for a period of 3 min. The reaction mixture contained 0.5 mL of 0.05 M Tris–SO_4_ buffer (pH: 7.4), 0.75 mL of *p*‐NPA (3 mM), 0.1 mL of distilled water, and 0.15 mL of the enzyme solution (final volume 1.5 mL). The control test did not contain enzyme in the test mixture. All measurements were performed as triplicates. The method was used to determine the inhibitory activities of the food additives studied.

### 2.7 Protein Determination

The amount of protein in the eluates during the affinity chromatography was determined by the modified Bradford method spectrophotometrically at 170–595 nm using bovine serum albumin for the construction of the standard graphs (Figures 1 and 2) [31].

### 2.8 SDS‐Polyacrylamide Gel Electrophoresis (SDS‐PAGE)

**Figure 3:**
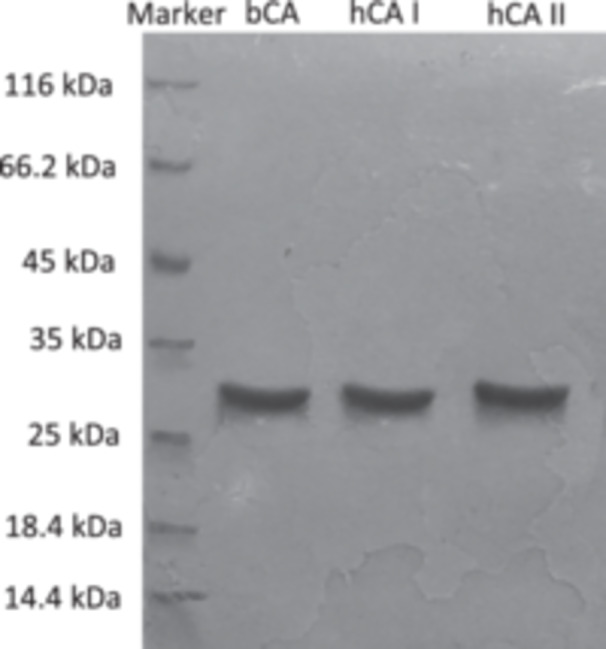
SDS‐PAGE image of the bCA and the isoenzymes hCAI and hCAII purified by affinity chromatography.

Enzyme purity was checked by SDS‐PAGE. The electrophoresis technique was applied according to Laemmli's method [32]. The running and stacking gels contained 10% and 5% acrylamide concentrations, respectively, also containing 10% SDS. 20 μL sample was applied to the electrophoresis medium. Gel staining was achieved with Coomassie brilliant blue R‐250 dye. The electrophoregram was photographed (Figure 3).

### 2.9 Preparation of Samples

Food additives from different companies were obtained from Trabzon Food Control Laboratory. Curcumin, saccharin, and butylated hydroxytoluene (BHT) was prepared by dissolving in dimethyl sulfoxide (DMSO) while other food additives were prepared by dissolving in pure water. The food additives used in the study are given in Table 1.

### 2.10 Determination of IC_50_ Values

To determine the effect of all samples on bCA, hCAI, and hCAII isoenzymes esterase activity at saturated substrate concentration, activity (%) ‐ inhibitor concentration [I] graphs were plotted. The inhibitor concentrations (IC_50_) which caused 50% inhibition from the equation of the curve in the plotted graphs were calculated.

### 2.11 Determination of K_i_ for Erythrosine B

To determine K_i_ values, three different inhibitor concentrations and five different substrate (*p*‐nitrophenyl acetate) concentrations (0.15–0.75 mM) were used. Lineweaver–Burk lines were drawn for each inhibitor concentration. In the graph equation, K_i_ values were calculated by using V_max_=V^I^_max_(1 + [I]/K_i_) formula for semi‐competitive and Noncompetitive inhibition.

### 2.12 In Silico ADME/Toxicology Analysis

Absorption, Distribution, Metabolism, and Excretion (ADME) and toxicology assessment were performed using SwissADME [33] for a total of 20 food additives. In ADME‐Toxicology evaluation, ligands were determined by Lipinski's rules, molecular weight, logP values, H‐bond acceptor, donor numbers, CYP2D6 inhibition, and blood‐brain barrier properties were investigated.

### 2.13 Statistical Analysis

All data are expressed as the mean of three replicates, and the regression analyses were done by using Microsoft Excel, with R^2^ over 0.98 in all data series.

## 3 Results

In this study, the bovine carbonic anhydrase enzyme (bCA) and human erythrocyte carbonic anhydrase isoenzymes (hCAI and hCAII) were purified by covalent binding of tyrosine after activation of the free hydroxyl groups of Sepharose 4B with cyanogen bromide (CNBr) and finally by diazotizing the sulfanilamide. Dialysis was performed to remove the buffer components from the purified enzymes. After that, concentrated protein solutions were obtained by the lyophilization process, which is a freeze‐drying method. hCAI was purified with a specific activity 4500.0 EU/mg proteins, yield of 49.85% from human erythrocytes. hCAII was purified with a specific activity of 7500.0 EU/mg proteins, yield of 47.08% from human erythrocytes. The specific activity for BCA was 16967 EU/mg proteins and was 968‐fold purified. SDS‐PAGE was applied to check the purity of the purified isoenzymes, and a single band was observed for each enzyme. Molecular weight of CA isoenzymes was found approximately 29 kDa (Figure 3). During the purification, the protein quantities in the eluates were determined both semi‐quantitatively and quantitatively. Semi‐quantitative protein determinations were based on the absorbance of each eluate at 280 nm. Quantitative protein determinations were performed by the Bradford method utilizing Coomassie‐Blue.

At the saturated substrate concentration, the inhibitory effect of twenty different food additives (12 colorants, 2 antioxidants, 3 sweeteners, 2 preservatives, and 1acidity regulator) on the esterase activity of purified bCA, hCAI, and hCAII isoenzymes were investigated. For this reason, esterase activity was measured at five different concentrations of each food additive. Activity (%)–[inhibitor] graphs were drawn for each food additive having an inhibitory effect. The inhibitor concentration values (IC_50_) which cause 50% inhibition were calculated from these graphs (Table 2). In addition, K_i_ value was calculated for Erythrosine B which showed a high inhibitory effect on the purified hCAI isoenzyme, and the inhibition type was determined.

**Table 2:** IC_50_ (μM) values of food additives studied by esterase activity method using bCA, hCAI, and hCAII enzymes.

Compound		IC_50_ (µM)^a^		
	ADI (mg/kg)	bCA	hCAI	hCAII
Patent Blue V	5	267.46 ± 0.09	316.98 ± 0.02	396.74 ± 0.03
Allura Red AC	7	762.12 ± 0.12	781.65 ± 0.06	504.04 ± 0.02
Tartrazine	7.5	659.67 ± 0.07	652.25 ± 0.05	621.83 ± 0.05
Ponceau 4R	0.7	239.84 ± 0.04	678.27 ± 0.03	617.09 ± 0.01
Sunset Yellow FCF	4	680.82 ± 0.05	350.05 ± 0.03	488.63 ± 0.07
Brilliant Blue FCF	6	136.26 ± 0.04	208.04 ± 0.03	364.30 ± 0.09
Brilliant Black BN	1	153.32 ± 0.04	91.14 ± 0.09	502.45 ± 0.03
Erythrosine B	0.1	10.85 ± 0.05	19.37 ± 0.04	5.22 ± 0.02
Amaranth	0.5	296.06 ± 0.06	496.04 ± 0.04	603.11 ± 0.11
Indigo Carmine	5	141.59 ± 0.07	1018.57 ± 0.04	242.03 ± 0.03
Curcumin	3	75.38 ± 0.09	189.10 ± 0.10	73.82 ± 0.02
Carminic Acid	2.5	586.92 ± 0.01	683.80 ± 0.00	581.07 ± 0.03
Saccharin	5	inactive	2049.10± 0.10	1015.22 ± 0.01
Acesulfame K	15	2350.50 ± 0.10	5997.71 ± 0.05	2872.21 ± 0.05
Aspartame	40	inactive	2816.87 ± 0.03	2935.79 ± 0.02
Citric Acid	NL	616.90 ± 0.10	1799.38 ± 0.03	1241.87 ± 0.07
Benzoic Acid	5	1850.70 ± 0.05	2784.04 ± 0.06	2988.81 ± 0.06
Sorbic Acid	25	1801.44 ± 0.04	4566.19 ± 0.06	3475.10 ± 0.10
Ascorbic Acid	NL	993.67 ± 0.03	1834.10 ± 0.10	2160.07 ± 0.07
Butylated Hydroxytoluene (BHT)	0.3	512.85 ± 0.05	462.89 ± 0.10	104.29 ± 0.09
Sulfanilamide	‐	4.93 ± 0.02	7.02 ± 0.01	8.02 ± 0.01

^a^ Mean from at least three determinations. Errors in the range of 3–5% of the reported value. ADI: acceptable daily intake NL: not limited.

The inhibition effect of food additives on the esterase activity of the bCA enzyme was studied for the first time in this study. IC_50_ values determined by bCA esterase activity method of twenty different food additives were between 10.85 ± 0.05–2350.50 ± 0.10 μM. When the investigated food additives were evaluated as in groups, the highest inhibition values were detected in the colorants, among which Allura Red AC showed the lowest inhibition, Erythrosine B, a synthetic colorant, showed the highest inhibition (IC_50(bCA)_: 10.85 ± 0.05 μM). The IC_50_ value of Erythrosine B (10.85 ± 0.05 μM) was found to have an IC_50_ value close to sulfanilamide, which is one of the most potent inhibitors of CAs. BHT (IC_50(bCA)_: 512.85 ± 0.05 μM) and ascorbic acid (IC_50(bCA)_: 993.67 ± 0.03 μM), which are used in most kinds of foods and beverages as antioxidant showed an inhibitory effect on the CA activity. Saccharin and aspartame as sweeteners had no effect on bCA activity. Also, Acesulfame K had the lowest inhibition (13% inhibition at 2 mg/mL concentration). The effect on CA activity of sorbic and benzoic acids, which were added to foods as preservatives, were found to be very low. Finally, IC_50(bCA)_ value of citric acid used as an acid regulator in the processing of foods was determined as 616.90 ± 0.10 μM (Table 2).

## 4 Discussion

In the literature, the effects of some food additives on hCAI and hCAII isoenzymes have been studied recently. This paper, for the first time, demonstrated the inhibition effect of several groups of food additives on hCAI and hCAII isoenzymes. The hCAI IC_50_ values of twenty different food additives determined by the esterase activity method are in the range of 19.37 ± 0.04–5997.71 ± 0.05 μM, while the hCAII IC_50_ values are in the range of 5.22 ± 0.02–3475.10 ± 0.10 μM. According to these results, the effect on hCAII is significantly higher, and these agents can be evaluated in the search for selective hCAII inhibitors to be used as potential drugs [33]. Considering the effect of food additives on both isoenzymes, it was determined that the colorants, especially the synthetic ones, had higher inhibition. Erythrosine B, which showed the highest inhibition in all three enzymes, should be studied further in new CA inhibitor drug research with new Erythrosine B derivatives, which are an oxy‐xanthene and benzoic acid derivative, similar to sulfanilamide derivatives. Esmaeili and colleagues studied the interaction between the colorant Allura Red AC with hCAI using different spectrophotometric techniques in vitro [22, 23]. The IC_50_ value obtained in that study was 565 μM, and it is consistent with our results. In addition, Sentürk and coworkers studied curcumin (IC_50(hCAI)_: 5.63 μM and IC_50(hCAII)_: 4.81 μM), a natural colorant [25]. Esimbekova et al. Recommend a series of single and very enzyme analysis systems to reveal the toxic effects of food preservatives such as sorbic acid, potassium sorbat, and sodium benzoate [34].

Results of our study indicated that sweeteners having little or no effect on bCA enzyme have a low inhibition effect on the activities of hCAI and hCAII isoenzymes (saccharin IC_50(hCAI)_: 2049.10 ± 0.10 μM, aspartame IC_50(hCAI)_: 2816.87 ± 0.03 μM and acesulfame K IC_50(hCAI)_: 5997.71 ± 0.05 μM; saccharin IC_50(hCAII)_: 1015.22 ± 0.01 μM, aspartame IC_50(hCAII)_: 2935.79 ± 0.02 μM and acesulfame K IC_50(hCAII)_: 2872.21 ± 0.05 μM). In the literature, saccharin shows low micromolar inhibition against hCAII, the isoform distributed in all human tissues [26]. The effect of citric acid on the bCA enzyme, which has nearly the same effect as the colorants do, is lower on both isoenzymes (IC_50(hCAI)_: 1799.38 ± 0.03 μM and IC_50(hCAII)_: 1241.87 ± 0.07 μM). The food preservatives sorbic acid and benzoic acid showed a lower inhibition on the isoenzymes.

In our study, K_i_ value was determined as 0.0028 ± 0.0011 mM for the purified hCAI isoenzyme for Erythrosine B, which showed the highest inhibition effect on all three enzymes, and inhibition type was found to be Noncompetitive (Table 3).

**Table 3:** K_i_ value and inhibition type of Erythrosine B showing inhibition effect on esterase activity of hCAI isoenzyme.

Inhibitor	IC_50_ (mM)^a^	[I] (mM)	K_i_ values^a^	Average K_i_ (mM)	Type of inhibition
		0.015	0.0029		
Erythrosine B	0.019	0.019	0.0017	0.0028	Noncompetitive
		0.023	0.0039		

^a^ Mean from at least three determinations. Standard errors in IC_50_ and K_i_ values are in the range of 3–5% of the reported value.

Recently, there has been a growing interest in the toxicity of the additives used in foods, especially azo dyes, and in the search for natural alternatives [35, 36, 37]. The main concern in the use of the synthetic coloring agents is the carcinogenic metabolites induced by azo reduction by the gut microbiota [38]. However, given the low absorption rates, the possibility of harming human health depends on the amount of colorant [39]. The research shows that especially after consumption of continuous use of synthetic food colorants, allergic reactions, rashes, skin swelling and hyperactivity, including a negative systematic effects on human health [40, 41]. In line with the approximations given by the Joint FAO/WHO Expert Committee on Food Additives, the maximum acceptable daily intake (ADI) of the additives used in the study are given in Table 2. The inhibitory effect of food additives on CA has been proven with the IC_50_ values given in Table 2. It should be noted that a 50% decrease in CA enzyme activity of the additives occurred in the presence of food additives at concentrations much lower than the daily intake (ADI) of food products. For example in the literatüre, although the acceptable daily intake for Allura Red is 7 mg/kg body weight, the in vivo comet assay performed by Shimado and colleagues showed that it can cause DNA damage in the glandular stomach, colon and lungs of mice [42]. In this context, new toxicity concerns have arisen for several synthetic colorants, such as Allura Red, which has the ability to bind to human serum albümin [43]. This study is related to its application as a drug rather than its application as a food additive. Numerous in vivo studies should be conducted on the molecular effects of food additives.

ADMET properties are very important clinically. To show drug properties, the molecule must meet the Lipinski rules. According to Lipinski rules; 1) the weight of the molecule must be less than 500 Da, 2) the number of hydrogen bond donors must be less than 5, 3) the number of hydrogen bond acceptors must be less than 10, 4) the MLogP value must be less than 4.15. ADMET screening results are given in Table 4. Accordingly, when drug similarities were examined, it was determined that 4 food additives (Allura Red AC, Sunset Yellow FCF, Indigo Carmine, and Curcumin) were found to comply with Lipinski rules. The most important reason for this result is molecular weights. The molecules except Allura Red AC, Sunset Yellow FCF, Indigo Carmine, and Curcumin have molecular weights greater than 500. In addition, Patent Blue V has a hydrogen bond acceptor count of more than 10 and an MLogP value of more than 4.15. Tartrazine, Ponceau 4 R, Brilliant Black BN, Amaranth, and Carminic acid molecules have a hydrogen bond count of more than 10. Brilliant Blue FCF and Erythrosine B molecules do not comply with Lipinski rules because their MlogP values exceed 4.15. Apart from these, the TPSA value indicates the surface width of molecules. This feature is important for the diffusion of the molecule into tissues and cell permeability. Being between 40 and 130 gives the molecule the ability to be a drug. Only 2 molecules (Erythrosine B, Curcumin) comply with this feature.

**Table 4:** Physicochemical descriptors, ADME parameters predictors, pharmacokinetic properties, druglike nature and medicinal chemistry friendliness of the food additives.

	Patent Blue V	Allura Red AC	Tartrazine	Ponceau 4 R	Sunset Yellow FCF	Brilliant Blue FCF	Brilliant Black BN	Erythrosine B	Amaranth	Indigo Carmine	Curcumin	Carminic acid
**Physicochemical Properties**												
Molecular weight (g/mol)	1159.43	496.42	534.36	604.47	452.34	792.85	867.68	879.86	604.47	466.35	368.38	492.39
Fraction Csp3	0.30	0.11	0.06	0.00	0.00	0.16	0.04	0.05	0.00	0.00	0.14	0.32
Num. rotatable bonds	18	5	6	5	4	12	10	0	5	3	8	3
Num. H‐bond acceptors	14	10	12	12	9	9	18	5	12	9	6	13
Num. H‐bond donors	2	1	0	1	1	0	2	0	1	2	2	9
TPSA (A^2^)	315.28	185.34	228.68	241.69	176.11	202.99	361.09	81.65	241.69	196.61	93.06	242.51
**Lipophilicity**												
Log *P*_o/w_ (iLOGP)	−55.59	−21.88	−39.04	−41.02	−24.67	−32.11	−60.85	−10.87	−39.87	−20.09	3.27	−0.87
Log *P*_o/w_ (XLOGP3)	7.65	2.42	0.44	2.09	2.09	4.80	2.34	6.02	2.64	0.22	3.20	0.53
Log *P*_o/w_ (WLOGP)	11.26	5.25	0.50	6.07	4.93	7.97	8.24	6.85	6.07	2.78	3.15	−1.52
Log *P*_o/w_ (MLOGP)	4.31	2.00	0.32	2.27	2.02	4.38	2.29	5.50	2.27	0.02	1.47	−2.95
Log *P*_o/w_ (SILICOS‐IT)	1.34	1.51	−0.81	0.34	0.92	2.31	0.27	7.21	0.34	0.78	4.04	−0.55
Consensus Log *P*_o/w_	−6.21	−2.14	−7.72	−6.05	−2.94	−2.53	−9.54	2.94	−5.71	−3.26	3.03	−1.07
**Water Solubility**												
[Log *S* (Ali)] S, MS, PS or IS	IS	MS	MS	PS	MS	PS	PS	PS	PS	S	MS	MS
**Pharmacokinetics**												
GI absorption	Low	Low	Low	Low	Low	Low	Low	Low	Low	Low	High	Low
BBB permeant	No	No	No	No	No	No	No	No	No	No	No	No
P‐gp substrate	Yes	Yes	Yes	Yes	Yes	Yes	Yes	Yes	Yes	Yes	No	Yes
CYP1A2 inhibitor	No	No	No	No	No	No	No	No	No	No	No	No
CYP2C19 inhibitor	No	No	No	No	No	No	No	No	No	No	No	No
CYP2C9 inhibitor	No	No	No	No	No	No	No	No	No	No	Yes	No
CYP2D6 inhibitor	No	No	No	No	No	No	No	No	No	No	No	No
CYP3A4 inhibitor	No	No	No	No	No	No	No	No	No	No	Yes	No
Log *K*_p_ (skin permeation) (cm/s)	−7.94	−7.61	−9.25	−8.50	−7.58	−7.73	−9.93	−7.39	−8.11	−8.99	−6.28	−8.93
**Druglikeness**												
Lipinski	No, 3 viol.	Yes, 0 viol.	No, 2 viol.	No, 2 viol.	Yes, 0 viol.	No, 3 viol.	No, 2 viol.	No, 2 viol.	No, 2 viol.	Yes, 0 viol.	Yes, 0 viol.	No, 2 viol.
Ghose	No, 4 viol.	No, 1 viol.	No, 1 viol.	No, 2 viol.	Yes	No, 4 viol.	No, 4 viol.	No, 3 viol.	No, 2 viol.	Yes	Yes	No, 2 viol.
Veber	No, 2 viol.	No, 1 viol.	No, 1 viol.	No, 1 viol.	No, 1 viol.	No, 2 viol.	No, 1 viol.	Yes	No, 1 viol.	No, 1 viol.	Yes	No, 1 viol.
Egan	No, 2 viol.	No, 1 viol.	No, 1 viol.	No, 2 viol.	No, 1 viol.	No, 2 viol.	No, 2 viol.	No, 1 viol.	No, 2 viol.	No, 1 viol.	Yes	No, 1 viol.
Muegge	No, 5 viol.	No, 1 viol.	No, 2 viol.	No, 3 viol.	No, 1 viol.	No, 2 viol.	No, 3 viol.	No, 2 viol.	No, 3 viol.	No, 1 viol.	Yes	No, 3 viol.
Bioavailability Score	0.17	0.55	0.17	0.17	0.55	0.17	0.17	0.17	0.17	0.55	0.55	0.11
**Medicinal Chemistry**												
Leadlikeness	No, 3 viol.	No, 1 viol.	No, 1 viol.	No, 1 viol.	No, 1 viol.	No, 3 viol.	No, 2 viol.	No, 2 viol.	No, 1 viol.	No, 1 viol.	No, 2 viol.	No, 1 viol.
Synthetic accessibility	7.68	3.36	4.06	3.57	3.07	5.40	4.49	4.17	3.57	3.28	2.97	5.04

	Saccharin	Acesulfame K	Aspartame	Citric acid	Benzoic acid	Sorbic acid	Ascorbic acid	Butylated hydroxytoluene (BHT)
Physicochemical Properties								
Molecular weight (g/mol)	183.18	201.24	294.30	192.12	122.12	112.13	176.12	220.35
Fraction Csp3	0.00	0.25	0.36	0.50	0.00	0.17	0.50	0.60
Num. rotatable bonds	0	0	9	5	1	2	2	2
Num. H‐bond acceptors	3	5	6	7	2	2	6	1
Num. H‐bond donors	1	0	3	4	1	1	4	1
TPSA (A^2^)	71.62	68.82	118.72	132.13	37.30	37.30	107.22	20.23
Lipophilicity								
Log *P*_o/w_ (iLOGP)	0.74	−4.51	1.59	−1.49	1.11	1.46	0.39	3.33
Log *P*_o/w_ (XLOGP3)	0.91	−0.34	−2.74	−1.72	1.87	1.33	−1.64	5.10
Log *P*_o/w_ (WLOGP)	0.82	0.77	−0.31	−1.25	1.38	1.20	−1.41	4.30
Log *P*_o/w_ (MLOGP)	−0.02	−0.73	0.27	−1.48	1.60	1.07	−2.60	4.12
Log *P*_o/w_ (SILICOS‐IT)	0.56	−0.39	0.54	−1.60	1.24	0.57	−1.15	4.34
Consensus Log *P*_o/w_	0.60	−1.04	−0.13	−1.51	1.44	1.13	−1.28	4.24
Water Solubility								
[Log *S* (Ali)] S, MS, PS or IS	VS	VS	HS	VS	S	VS	VS	MS
Pharmacokinetics								
GI absorption	High	High	High	Low	High	High	High	High
BBB permeant	No	No	No	No	Yes	Yes	No	Yes
P‐gp substrate	No	No	No	No	No	No	No	No
CYP1A2 inhibitor	No	No	No	No	No	No	No	No
CYP2C19 inhibitor	No	No	No	No	No	No	No	No
CYP2C9 inhibitor	No	No	No	No	No	No	No	No
CYP2D6 inhibitor	No	No	No	No	No	No	No	Yes
CYP3A4 inhibitor	No	No	No	No	No	No	No	No
Log *K*_p_ (skin permeation) (cm/s)	−6.77	−7.77	−10.04	−8.69	−5.72	−6.04	−8.54	−4.02
Druglikeness								
Lipinski	Yes, 0 viol.	Yes, 0 viol.	Yes, 0 viol.	Yes, 0 viol.	Yes, 0 viol.	Yes, 0 viol.	Yes, 0 viol.	Yes, 0 viol.
Ghose	No, 1 viol.	No, 2 viol.	Yes	No, 2 viol.	No, 3 viol.	No, 3 viol.	No, 2 viol.	Yes
Veber	Yes	Yes	Yes	Yes	Yes	Yes	Yes	Yes
Egan	Yes	Yes	Yes	No, 1 viol.	Yes	Yes	Yes	Yes
Muegge	No, 1 viol.	No, 1 viol.	No, 1 viol.	No, 1 viol.	No, 1 viol.	No, 1 viol.	No, 1 viol.	No, 2 viol.
Bioavailability Score	0.55	0.85	0.55	0.56	0.85	0.85	0.56	0.55
Medicinal Chemistry								
Leadlikeness	No, 1 viol.	No, 1 viol.	No, 1 viol.	No, 1 viol.	No, 1 viol.	No, 1 viol.	No, 1 viol.	No, 2 viol.
Synthetic accessibility	2.25	3.15	2.85	2.18	1.00	2.79	3.47	1.48

Abbreviations: BBB, blood brain barrier; GI, gatrointestinal; HS, highly soluble; IS, insoluble; MS, medium soluble; PS, poorly soluble; P‐gp, P‐glycoprotein; S, soluble; TPSA, topological polar surface area; VS: very soluble.

When the pharmacokinetic properties of the molecules are considered, the only molecule with high gastrointestinal absorption is curcumin. The fact that all other molecules have the property to bind to P‐glycoprotein, and to be a substrate, reveals the reason why the gastrointestinal absorption of the different molecules is low. Since the molecules cannot pass any blood‐brain barrier, they do not have the opportunity to be drug precursors, especially for the treatment of central nervous system diseases. In addition, when the molecules were examined in terms of CYP family inhibition, it was seen that only curcumin could cause CYP2C9 and CYP3A4 inhibition. This is an undesirable situation for medicinal chemistry. It indicates that this molecule may interact with different drugs and that it may exhibit characteristics to bind many proteins. As a result, it has been revealed that although only curcumin has the characteristics of being a drug precursor, it can also be a drug molecule with low specificity due to its affinity to the CYP family.

## 5 Conclusions

The information obtained in this study revealed two important aspects of molecules that are components of products consumed as food and beverage. The first of these is the problems that can be caused by the inhibition of carbonic anhydrase, which performs an important function for the elimination of the metabolic end‐product carbon dioxide (CO_2_) in all human cells. Whether this unwanted situation is important for the body should be demonstrated with in vivo studies on absorption and tissue distribution of food additives. In fact, the relationship between CA inhibition and diseases increasing parallel to ready‐to‐use food consumption should be revealed. The possible inhibition of carbonic anhydrase enzymes of the beneficial microorganisms in gastrointestinal tract by the CA inhibiting food additives may also cause serious health problems. Therefore, the use of the food addives with no or low CA inhibition should be recommended in food production, and new additives should be sought for if the additives with low CA inhibition are not available for certain purposes. The second is that the production of novel carbonic anhydrase inhibitors with clinically important drug potential starting from the pure substances studied is possible. In this sense, it will be a good starting point with the additives studied, especially Erythrosine B, whose inhibitory effect is close to the standard inhibitors.

## Author Contributions

**Ayça Aktaş Karaçelik:** writing – original draft, visualization, project administration, methodology, investigation, formal analysis, data curation, conceptualization. **Murat Küçük:** writing – review and editing, visualization, validation, project administration, methodology, investigation, formal analysis, data curation. **Semra Alkan Türkuçar:** writing – review and editing, investigation, data curation. **Şükrü Beydemir:** writing – review and editing, investigation, data curation.

## Ethics Statement

The study design was approved by the scientific research ethics committee of Karadeniz Technical University, Faculty of Medicine Scientific Research Ethics Committee, Trabzon, Türkiye.

## Conflicts of Interest

The authors declare no conflicts of interest.

## Data Availability

The data that support the findings of this study are available from the corresponding author upon reasonable request.
